# Association for Promoting Employment of the Defective

**Published:** 1908-09-12

**Authors:** P. S. G. Propert, Beatrice Mary Chamberlain, Arthur Clay, E. A. Eardley-Wilmot, Percy Gates, Bertha James, R. O. B. Lane, E. Noel Walker, G. E. Shuttleworth, Pauline D. Townsend, B. C. Arnould

**Affiliations:** President.; Chairman; Hon. Secretary


### Editor's Letter-Box.

### ASSOCIATION FOR PROMOTING EMPLOYMENT OF THE DEFECTIVE.

(*To the Editor of* The Hospital.)

Sir,—Two years ago you kindly aided us by inserting an appeal for funds needed for employment rooms and office. We should be much obliged if you would help us in making known that a house and garden suitable for these purposes has now been obtained by the Association, and will be opened in a few weeks' time.

The Association was formed in 1901 to promote the protection and employment of those living in Fulham, Kensington, Chelsea, and Hammersmith, who being mentally or physically defective—*i.e.* feeble-minded, imbecile, epileptic, crippled, invalids, deaf or blind, need special care. Since that time many names have been referred of those leaving the L.C.C. special schools for defective children, and of others, children and adults who need help or supervision. Some have been helped to find employment or encouraged by the certificates given to those who keep their places. Each year specimens of homework, toys, bookbinding, etc., produced by invalids and cripples have been shown at the exhibition of the Home Arts and Industries Association, and gained certificates for workmanship and design. Children unable to attend special schools have been visited, and, when beyond the care of their relations, referred if possible to suitable institutions. Meetings have also been held for mothers of defective children.

It is now proposed to use the house obtained for manual occupation—wood-work, gardening, etc.—for feeble-minded boys over school age when out of work; for needle-work and housewifery classes for girls; for kindergarten lessons for children unable to attend school; and as an office, and *depôt* for the sale of homework, toys, etc., by invalids. It is hoped that the house will become a local centre for those who, in various ways, are brought in contact with the defective, and who desire to give time and thought to helping them where information about suitable employment, handicrafts, etc., may be obtained. We may add that the scheme has the support of many with long experience of the class the Association desires to help, who realise that, whatever results may follow the report of the Royal Commissions on the Feeble-minded and on the Poor-law, much must still depend on voluntary efforts to promote their welfare with the aid of their relations and private charity.

It is estimated that £200 per annum will be needed to carry on the work of the Association. We earnestly appeal to residents in the boroughs of Kensington, Fulham, Chelsea, and Hammersmith to aid this effort to give a helping-hand to those living around them who are exposed to special difficulties and dangers.

Any willing to help by donations or subscriptions, or by gifts of furniture suitable for office and work-rooms, are asked to communicate with the Treasurer, Sir Edward Noel Walker, K.C.M.G., 52 Warwick Road, Kensington, or with the Hon. Secretary, Miss Arnould, 9 Nevern Square, Earl's Court.

Llangattock, President.
P. S. G. Propert, Chairman.
Beatrice Mary Chamberlain.
Arthur Clay.
E. A. Eardley-Wilmot.
Percy Gates.
Bertha James.
R. O. B. Lane.
E. Noel Walker.
G. E. Shuttleworth, M.D.,
Pauline D. Townsend.
B. C. Arnould, Hon. Secretary.

### THE MEDICAL DIRECTORY.

(*To the Editor of* The Hospital.)

